# Das Cannabinoid-Hyperemesis-Syndrom – ein narratives Review

**DOI:** 10.1007/s00115-025-01864-0

**Published:** 2025-07-21

**Authors:** Udo Bonnet

**Affiliations:** 1 Klinik für Seelische Gesundheit, Evangelisches Krankenhaus Castrop-Rauxel, Akademisches Lehrkrankenhaus der Universität Duisburg-Essen, Grutholzallee 21, 44577 Castrop-Rauxel, Deutschland; 2 LVR-Universitätsklinik Essen, Klinik für Psychiatrie und Psychotherapie, Medizinische Fakultät, Universität Duisburg-Essen, Essen, Deutschland

**Keywords:** Cannabisabhängigkeit, THC, Zyklisches Erbrechen, Haloperidol, Capsaicin, Cannabis dependence, THC, Cyclic hyperemesis, Haloperidol, Capsaicin

## Abstract

**Hintergrund:**

Bis vor 25 Jahren waren zyklische Brechattacken unter chronischem Cannabiseinfluss nahezu unbekannt. Nach der Legalisierung nichtmedizinischen Cannabis in Nordamerika und der damit einhergehenden Zunahme des Cannabiskonsums, einschließlich hochpotenter Sorten steigt dort die Zahl der Patienten mit cannabisbezogenem zyklischem Erbrechen. Die ROME-IV-Kriterien der Rome Foundation definieren das cannabisinduzierte zyklische Erbrechen inzwischen als Cannabinoid-Hyperemesis-Syndrom (CHS). Dieses Review möchte über das CHS informieren, da auch in Deutschland nach der Legalisierung mit einer Zunahme der Fälle zu rechnen ist.

**Methode:**

Selektive Übersichtsarbeit.

**Ergebnisse:**

Das CHS wird am häufigsten in den Notaufnahmen registriert. Eine eindeutige Differenzierung von CHS und Cyclic-vomiting-Syndrom (CVS), bei dem ein Drittel der Betroffenen ebenfalls regelmäßig Cannabis konsumiert, ist nur durch Feststellung einer Vollremission während einer 6‑ bis 12-monatigen Cannabisabstinenz möglich. Daher werden in den Notaufnahmen zunächst Mischformen aus CVS und CHS gesehen (suspektes CHS), auch mit versteckten vital bedrohlichen abdominellen Komorbiditäten. Das schwere Erbrechen kann ebenfalls zu gravierenden Komplikationen führen. Übliche Antiemetika helfen oft nicht. Heißes Duschen und Baden sowie Haloperidol i.m. (5 mg) können das schwere Erbrechen akut linderen. Auch eine Einreibung des Abdomens mit 0,1 %iger Capsaicincreme wirkt antiemetisch, jedoch weniger schnell.

**Diskussion:**

In ROME-IV gilt das CHS nosologisch als spezielle Variante des CVS. Es ist insbesondere eine spezifische cannabisbezogene oft schwere körperliche Erkrankung. Da Heilung nur durch eine anhaltende Cannabisabstinenz zu erreichen ist, ist das suspekte CHS in den Notaufnahmen eine interdisziplinäre Herausforderung für die Gastroenterologie, Neuropsychiatrie und das Suchthilfesystem.

**Zusatzmaterial online:**

Zusätzliche Informationen sind in der Online-Version dieses Artikels (10.1007/s00115-025-01864-0) enthalten.

## Abstract (en)

**Background:**

Until 25 years ago cyclic vomiting attacks under the chronic influence of cannabis were virtually unknown. Following the legalization of non-medical cannabis in North America and the associated increase in cannabis use, including high-potency strains, the number of patients there with cannabis-related cyclic vomiting is increasing. The ROME-IV criteria now define cannabis-induced cyclic vomiting as cannabinoid hyperemesis syndrome (CHS). This review aims to provide information about CHS, as an increase in cases is also to be expected in Germany following legalization.

**Method:**

Selective review article.

**Results:**

The CHS is most frequently registered in emergency departments. A clear differentiation between CHS and cyclic vomiting syndrome (CVS), in which one third of those affected also regularly use cannabis, is only possible by establishing full remission during a 6–12 month cannabis abstinence. Therefore, mixed forms of CVS and CHS are initially seen in emergency departments (suspected CHS), also with hidden life-threatening abdominal comorbidities. Severe vomiting can also lead to serious complications. The commonly used antiemetics often do not help. Hot showers and baths as well as i.m. haloperidol (5 mg) can provide acute relief from severe vomiting. Rubbing the abdomen with 0.075–0.1% capsaicin cream also has an antiemetic effect but less quickly.

**Discussion:**

In ROME-IV the CHS is nosologically considered a special variant of CVS. In particular, it is a specific cannabis-related, often severe physical disorder. As healing can only be achieved through sustained cannabis abstinence, suspected CHS in emergency departments is an interdisciplinary challenge for gastroenterology, neuropsychiatry and the addiction support system.

Bis vor 25 Jahren war ein schweres zyklisches Erbrechen im Verlauf einer ausgeprägten Cannabisabhängigkeit nahezu unbekannt. Mit der zunehmenden Verfügbarkeit auch hochpotenter Cannabispräparate in den Ländern, die den nichtmedizinischen Konsum von Cannabis legalisiert haben, ist dort die Häufigkeit des zyklischen Cannabis-Hyperemesis-Syndroms (CHS) angestiegen. Dieses ist nun auch in Deutschland zu erwarten.

Cannabis (*Cannabis sativa* L.) gehört zu den weltweit am weitesten verbreiteten Rauschmitteln und enthält über 500 Wirkstoffe. Darunter sind über 100 Phytocannabinoide, eine Gruppe von Terpenophenolen (oder Meroterpenoide), von denen einige an Cannabinoid-1- und/oder -2-Rezeptoren (CB1- und CB2-R) des Endocannabinoidsystems binden [30]. Delta-9-Tetrahydrocannabidiol (THC) und Cannabidiol (CBD) sind die bekanntesten, wobei CBD im Gegensatz zu THC nicht psychoaktiv ist und nicht die CB1‑R stimuliert [30]. Der Cannabisrausch entsteht hauptsächlich über die Aktivierung kortikaler und limbischer CB1‑R durch THC [5, 30]. Dessen antiemetische Eigenschaften helfen auch gegen chemotherapieinduziertes Erbrechen [5, 15, 30]. Bei vielen psychoaktiven Substanzen sind paradoxe Reaktionen bekannt [7].

Bis vor 25 Jahren waren cannabisbezogene Brechattacken außerhalb des Erstkonsums, schweren Intoxikation oder Entzugssyndromen eine absolute Rarität und wurden meistens auf Kontaminationen zurückgeführt [5]. Im Jahre 2004 wurden erstmals schwere zyklisch verlaufende Brechattacken im Zusammenhang mit langjährigem Cannabiskonsum in Australien berichtet [2]. Aufsehenerregend waren Berichte, dass Betroffene die schweren Brechattacken durch heißes Duschen oder Baden dämpfen konnten [2, 5, 14]. Diese Beschwerden wurden inzwischen bei den diagnostischen Kriterien für funktionelle gastrointestinale Störungen (ROME IV, [20]) als Cannabinoid-Hyperemesis-Syndrom (CHS) definiert (eTabelle 1). Dabei wurde berücksichtigt, dass das CHS auch beim nichtmedizinischen Konsum synthetischer CB1-R-hochaffiner Cannabinoide als auch unter medizinischem Nabilon und Dronabinol beobachtet wurde. Auch Delta-8-THC und hochdosiertes CBD werden verdächtigt [4, 10, 14, 21].

In den Ländern mit hohem Cannabiskonsum USA, Kanada, Neuseeland und Australien kommen vermehrt Patienten mit CHS in die Notaufnahmen der Krankenhäuser, auch in die pädiatrischen [14–17]. Besonders auffällig ist ein zeitlicher Zusammenhang mit der Legalisierung des nichtmedizinischen Cannabiskonsums [14–17]. Kürzlich wurde auch in der *NYTimes* darüber berichtet [26]. In Nordamerika soll inzwischen bei etwa 5 % der chronischen Konsumenten nichtmedizinischen Cannabis ein CHS existieren [16, 17]. Auch die französische Suchtüberwachung findet seit 10 Jahren zunehmend cannabisbezogene zyklische Brechattacken [8, 28]. In Großbritannien soll die aktuelle Prävalenz in der Bevölkerung bei 0,2 % liegen im Vergleich zu 0 % in einem gemeinsamen Datensatz aus 25 Ländern inklusive Deutschland [12]. Das könnte bedeuten, dass das CHS in Europa noch unterdiagnostiziert ist. In Deutschland scheint die Existenz des CHS noch zu wenig bekannt zu sein, sowohl bei Cannabiskonsumenten, dem Suchthilfesystem als auch bei Ärzten [4, 5]. Es gibt Berichte über Betroffene, die schon viele Krankenhausaufenthalte mit wiederholt aufwendiger Diagnostik hatten, bevor ein Zusammenhang zwischen den zur Aufnahme führenden schweren Brechattacken und Cannabis vermutet wurde [4]. Jedoch steigt die Aufmerksamkeit in den Notaufnahmen mit ersten Leitlinien [13, 25].

Dieses Review möchte über das CHS informieren, über die Schwierigkeiten der Diagnostik, die Komplikationen und den aktuellen Stand zur Behandlungsmöglichkeiten, Prognose und Pathogenese.

## Methodik

Es erfolgte eine selektive Literaturrecherche in der PubMed Datenbank (06/2025).

## Ergebnisse

### Begleiterscheinungen, Komplikationen und Differenzialdiagnosen des CHS

eTabelle 2 zeigt die bisher bekannten Begleiterscheinungen und Komplikationen des CHS. Bisher wurden 8 Todesfälle (alle USA) im Kontext von CHS beschrieben: meistens maligne Herzrhythmusstörungen. Allerdings konnte hier eine Beteiligung von QTc-Intervall-verlängernden Begleiterkrankungen, Medikamenten oder Drogen nicht sicher ausgeschlossen werden [18, 26]. Bei eingeschränkter CHS-Bekanntheit sind wahrscheinlich einige Todesfälle „verborgen“.

In eTabelle 3 sind die wichtigsten Differenzialdiagnosen zusammengefasst. Die größte diagnostische Herausforderung liegt darin, zwischen CHS und Cyclic-vomiting-Syndrom (CVS) zu differenzieren, das identische Symptome wie das CHS haben kann, jedoch während der Cannabisabstinenz nicht verschwindet [6, 14, 20, 21, 27]. Etwa ein Drittel der Jugendlichen und jungen Erwachsenen mit CVS betreiben auch einen regelmäßigen Cannabiskonsum, den sie zur Symptomlinderung oder aus hedonistischen Gründen einsetzen [11]. Wenn andere Differenzialdiagnosen ausgeschlossen sind, sollten daher die Fälle, die sich erstmals in einer Notaufnahme wegen cannabisbezogenem zyklischem Erbrechen vorstellen zunächst als Mischformen aus CVS und CHS eingeschätzt werden (suspektes CHS; [6]).

### Erweiterte Diagnostik und Verlauf

Das CHS kommt hauptsächlich bei jungen Erwachsenen vor, die bekanntlich am häufigsten nichtmedizinisches Cannabis konsumieren (aber auch Berichte ab dem 11. LJ; [14, 15]). In eTabelle 3 wird deutlich, dass sich hinter der Symptomatik eines CHS auch vital bedrohliche Erkrankungen verstecken können. Deshalb sollte ein Anfangsverdacht auf ein CHS zunächst gründlich abgeklärt werden, besonders, wenn folgende *Risikofaktoren für einen komplizierten Verlauf oder einer vitalbedrohlichen Mimikry* vorliegen:

- Patienten über dem 50. Lebensjahr,
- Fehlen des typischen zyklischen Verlaufs,
- kontinuierliche Verschlechterung,
- mehrere kardiovaskuläre Risikofaktoren.

Neben dem Drogenurin und Routinelabor inklusive Troponin und NT-BNP sollten gastroenterologische und suchtmedizinische Untersuchungen erfolgen [13, 25]. Zusätzlich können ein Schwangerschaftstest, eine zerebrale Bildgebung, eine Thoraxröntgenuntersuchung sowie psychiatrisch-psychosomatische, neurologische, kardiologische, angiologische oder nephrologische Untersuchungen richtungsweisend sein ([13, 25]; eTabelle 3).

Je häufiger die zyklischen Brechattacken beim CHS auftreten, desto heftiger können sie und desto kürzer die dazwischenliegenden symptomfreien Intervalle werden [5, 14, 24]. Das gilt allerdings auch für das CVS, das in allen Altersklassen vorkommt, jedoch auch eine relevante Komorbiditätslast, z. B. bezüglich der Migräne hat [27]. Beim CHS findet der Enzymimmunoassay (üblicher Bedside-Test) typischerweise einen THC-COOH-Wert über 100 ng/ml im Urin, als Hinweis auf einen chronischen Cannabiskonsum [9]. Dieser Wert persistiert oft über 4 bis 8 Wochen nach Konsumstopp (*eigene Beobachtung*).

### Behandlung

Nichtmedikamentös wird von Betroffenen in der Regel heißes Duschen oder Baden eingesetzt, um die schweren Attacken zu mildern, was jedoch nicht nachhaltig genug ist und bei zu langer Nutzung zu Komplikationen führt (eTabelle 2). Klassische Antiemetika sind oft wirkungslos [5, 21]. Opioiderge Analgetika helfen allenfalls gegen die begleitenden epigastrischen Schmerzen und eine häufigere Anwendung sollte wegen des eigenen Abhängigkeitspotenzials bei Cannabiskonsumenten vermieden werden. Synthetische und medizinische Cannabinoide verschlechtern pathognomonisch das CHS [4].

Die beste Evidenz zur Kompensation des akuten suspekten CHS liegt aktuell für die parenterale Gabe der starken Dopamin-2-Rezeptor-Antagonisten Haloperidol (0,05 mg/kg) oder Droperidol (2,5 mg) vor, bei jedoch eingeschränkter Tolerabilität (manchmal akute Dystonien; [21, 23]) und noch niedriger Vertrauenswürdigkeit nach GRADE (*eigene Einschätzung*). Auch die topische abdominelle Applikation einer 0,1 %igen Capsaicincreme ist wirksam, jedoch weniger schnell [19, 21, 23] und noch sehr niedriger bis niedriger Vertrauenswürdigkeit nach GRADE (*eigene Einschätzung*). Anekdotisch werden in der Akutsituation positive Behandlungserfahrungen mit parenteralem Lorazepam oder Scopolamin-Pflaster berichtet [13, 14, 21]. In der phasenprophylaktischen Behandlung wird von manchen Autoren zu oralen trizyklischen Antidepressiva wie Amitriptylin oder Doxepin geraten, die auch als „First-line“-Prophylaxe bei CVS eingesetzt werden [21, 27]. Für das CHS fehlt hier jedoch noch jegliche Evidenz.

Einzig und allein ist eine Heilung des CHS nur durch anhaltende Cannabisabstinenz zu erreichen [6]. Deshalb sollte jeder Verdachtsfall idealerweise über den neurologischen oder psychiatrischen Konsiliardienst zur Abstinenzeinleitung motiviert und soziotherapeutische Wege vermittelt werden. Ohne professionelle Hilfe erreichen nur die wenigsten Betroffenen eine nachhaltige Abstinenz (s. Prognose). Anhand der oben genannten Erfahrungen und Evidenz wird die Behandlungsempfehlung in eTabelle 4 gegeben, die sich an bisherige Leilinien [13, 25] anlehnt.

### Prognose

Prospektive Beobachtungsstudie aus den USA zeigen, dass die meisten Patienten, die wegen cannabisbezogenen schweren zyklischen Erbrechens in die Notaufnahmen kamen, nach der Entlassung weiter Cannabis konsumierten, obwohl sie wussten, dass Cannabis für ihr Erbrechen verantwortlich sein kann [29]. Nur 13 % wendeten sich an die Suchthilfe. Ein Viertel kam innerhalb von 3 Monaten wiederholt in die Notaufnahmen [29]. Durch D2-Dopamin-Rezeptor-Antagonisten und topisches Capsaicin kann nur akut eine Symptomreduktion erreicht werden [19, 21, 23]. Es gibt Berichte, bei denen schwer Betroffene ihre Brechattacken durch anhaltendes heißes Baden unterdrücken, um weiter in der Badewanne Cannabis konsumieren zu können [5].

Eine Vollremission bzw. Heilung des CHS ist nur durch stabile Abstinenz von Cannabis möglich wie zwei Kasuistiken mit langjähriger Symptomfreiheit zeigen: Follow-up 5 bzw. 9 Jahre (2 *definitiv valide CHSe;* [6]). In einer Fallserie mit 98 Betroffenen gab es 10 Fälle mit längerer Nachbeobachtung: Nach 3 Monaten waren die 3 Patienten mit fortlaufendem Cannabiskonsum nicht beschwerdefrei, anders als die 7 Abstinenten. Etwa 1 Monat nach Konsumstopp hatten letztere keine Entzugssymptome und kein CHS mehr [24].

### Pathogenese

Die „Emergenz“ des CHS läuft parallel zur Verfügbarkeit psychoaktiv deutlich stärkerer Cannabissorten als vor dem Jahr 2000 [2, 5, 14, 21]. Nach langjährigem, dann auch hochdosiertem/potentem Cannabiskonsums soll nach Manifestation einer gesteigerten CHS-Vulnerabilität bei weiterem Konsum eine Chronifizierung dieser entstehen [5]. Erste Kandidatengene sind identifiziert, die eine Vulnerabilität für zyklisches Erbrechen unter chronischem Cannabis bahnen könnten [22]. Bezüglich der weiteren „Vulnerabilisierung“ gibt es weitere Hypothesen:

1. *Sensitisierung des zentralen Brechzentrums*: Im Bereich der Area postrema entsteht durch den längeren Einfluss höherer THC-Mengen oder deutlich CB1-R-affine synthetische Cannabinoide eine vagusvermittelte schrittweise Absenkung der Schwelle zur toxischen Reaktion auf diese Substanzen inklusive Emesis [5, 21]. Die D2-Rezeptor-Antagonisten wirken direkt „schwellenerhöhend“ [21, 23].
2. *Endocannabinoid-Hypothese*: Der chronische langfristig hochdosierte Cannabiskonsum verändert das periphere (gastrointestinale) und zentrale Endocannabinoidsystem und führt zur Sensitisierung der emetischen Reaktion [10]. Über einen eingebundenen 5‑HT1A-Rezeptor-vermittelten Mechanismus könnte auch CBD hier emetisch wirken [10].
3. *Autonomes Nervensystem und Hypothalamus-Hypothese*: Die Linderung des Erbrechens durch heißes Wasser oder Capsaicin auf abdominelle Hautregionen favorisiert einen kurzfristig „desensitisierenden“ Einfluss peripherer Transient-receptor-potential-vanilloid 1(TRPV1)-Rezeptoren, die mit dem gastrointestinalen Endocannabinoidsystem interagieren. Über diesen Mechanismus könnte das sympathische Nervensystem [1] die Schwelle zum Erbrechen im Brechzentrum vorübergehend heraufregulieren. Die zyklische Komponente soll über einen endocannabinoidmodulierten Einfluss des Hypothalamus auf die emetische Schwelle unter CB1-R-Agonisten (*zyklische Sensitisierung*) entstehen [5, 10].
4. *Vasodilatation-Hypothese*: zunehmende cannabisinduzierte Gefäßerweiterung im Splanchnikusgebiet, was über einen Steal-Effekt Übelkeit auslösen könnte [5, 24].
5. *Andere Faktoren*: In einem sicheren CHS-Fall wurde eine Pseudoobstruktion im Dünndarm beschrieben [4]. Vulnerabilisierte (hochregulierte/sensitisierte) Growth-differentiation-factor-15-Rezeptoren in der Area postrema analog zur phänomenologisch ähnlichen Hyperemesis gravidarum?

## Diskussion und Zusammenfassung

Dieser Artikel soll das Krankheitsbild und das Management des CHS noch geläufiger machen. Die Zunahme der Fälle in Nordamerika wurde vermutlich durch einen nach der Legalisierung zunehmenden nichtmedizinischen Cannabiskonsum, auch potenterer Sorten ermöglicht [14–17]. Vermutlich sind viele CHS-Fälle noch unter der Diagnose eines CVS, wie die unterschiedliche Prävalenzen des CVS in den USA (2 %) und Canada (1 %) andeuten [3, 12]. Erst eine Abstinenzzeit von 6 bis 12 Monaten ohne Durchbruch zyklischer Brechattacken ist Voraussetzung für eine sichere CHS-Diagnose [6]. Alle anderen Fälle sind bei Cannabiskonsumenten zunächst suspekte CHS-Diagnosen [6]. Alle kontrollierten klinischen Studien zum CHS wurden bisher in Notaufnahmen durchgeführt und behandelten sehr wahrscheinlich Mischpopulationen aus CVS und CHS [9, 14, 19, 29].

## Fazit für die Praxis

- Leitsymptome des Cannabinoid-Hyperemesis-Syndroms (CHS) sind schweres zyklisches Erbrechen und Linderung durch heißes Duschen und Baden bei chronischen Cannabiskonsumenten.
- Eine Zunahme des CHS ist ein Marker für die Zunahme des Cannabiskonsums nach der Legalisierung nichtmedizinischen Cannabis in Nordamerika.
- Die häufigsten Differenzialdiagnosen des CHS sind Migräne und das Cyclic-vomiting-Syndrom (CVS), wobei das CVS und Migräne nicht selten zusammen vorkommen.
- Bei Cannabiskonsumenten kann das CHS vom CVS eindeutig nur durch eine Cannabisabstinenz von 6 bis 12 Monaten differenziert werden, in denen keine zyklischen Brechattacken mehr auftreten (*sicheres CHS*). Bis dahin gilt es als *suspektes CHS*.
- Das suspekte CHS kann lebensbedrohliche Komplikationen haben und lebensbedrohliche Komorbiditäten maskieren. Deshalb sollte es einmal gründlich stationär körperlich und neuropsychiatrisch abgeklärt werden.
- Bei CHS findet sich nach permanentem Konsumstopp oft wochenlang eine THC-COOH-Konzentration über 100 ng/ml.
- Eine Vollremission bzw. Heilung des CHS ist nur durch anhaltende Abstinenz von Cannabis möglich.

## Supplementary Information

eTabelle 1: Diagnosekriterien des Cannabis Hyperemesis Syndroms (CHS)* (ROME IV, B3c.) [20]

eTabelle 2: Begleiterscheinungen und Komplikationen des CHS [5, 15, 21, 24, 27]

eTabelle 3: Differenzialdiagnosen des CHS* [5, 15, 21, 24, 27]

eTabelle 4: Behandlungsempfehlung [5, 6, 13, 14, 21, 25]
