# Supplementary material for: The cannabinoid hyperemesis syndrome—A narrative review
Source: Nervenarzt. 2025 Jul 21;97(4):377–81. [Article in German] doi: 10.1007/s00115-025-01864-0 (PMC13314702; doi:10.1007/s00115-025-01864-0)
Supplement: Supplementary file 1 — eTabelle 1: Diagnosekriterien des Cannabis Hyperemesis Syndroms (CHS)* (ROME IV, B3c.) [20] [file 115_2025_1864_MOESM1_ESM.docx]

eTabelle 1: Diagnosekriterien des Cannabis Hyperemesis Syndroms (CHS)* (ROME IV, B3c.) (20)

| Alle folgenden Kriterien müssen erfüllt sein (für die letzten 3 Monate nach Symptombeginn seit mindestens 6 Monaten vor der Diagnose): |
| --- |
| - Stereotypes episodisches (zyklisches) Erbrechen |
| - Auftreten nach längerem Cannabiskonsum |
| - Verschwinden bei nachhaltiger Abstinenz |
| Unterstützendes Kriterium: Pathologisches Bade-Verhalten (längere heiße Bäder und Duschen) |
| *weitere Charakteristika: meistens Alter < 50 Jahre, Unwirksamkeit klassischer Antiemetika (5,21) |
