# Supplementary material for: The cannabinoid hyperemesis syndrome—A narrative review
Source: Nervenarzt. 2025 Jul 21;97(4):377–81. [Article in German] doi: 10.1007/s00115-025-01864-0 (PMC13314702; doi:10.1007/s00115-025-01864-0)
Supplement: Supplementary file 2 — eTabelle 2: Begleiterscheinungen und Komplikationen des CHS [5, 15, 21, 24, 27] [file 115_2025_1864_MOESM2_ESM.docx]

eTabelle 2: Begleiterscheinungen und Komplikationen des CHS (5,15,21,24,27)

| *Begleiterscheinungen der zyklischen Brechattacken* |
| --- |
| - Epigastrische Schmerzen (immer) |
| - Übelkeit (immer) |
| - Fatigue (meistens) |
| - Erhöhter Sympathikotonus mit leichter Sinustachykardie, arterieller Hypertonie, Leukozytose bzw. CRP-Erhöhung, Gastritis, Refluxösophagitis (meistens) |
| - Leichte bis moderate CK-Erhöhung (meistens) |
| - Schwitzen (Dehydration, Elektrolytverlust inklusive Magnesium) |
| *Komplikationen des zyklischen schweren Erbrechens* |
| - Zahnerosionen bis zum Zahnverlust (meistens) |
| - Appetit- und Gewichtsverlust (meistens) |
| - Periorbitale Petechien (selten) |
| - Aspirationspneumonie (selten) |
| - Elektrolytverlust (*meistens*)* – typischerweise Hypokaliämie, Hyponatriämie, Hypochlorämie mit metabolischer Alkalose; vereinzelt Hypophosphatämie. |
| - Wernicke Enzephalopathie, zentrale pontine Myelinolyse |
| - Dehydration (*meistens*)** |
| - Mallory Weiss-Syndrom, Ösophagusperforation mit Pneumomediastinum (*selten*) |
| *Komplikationen des pathologischen Badens* |
| - Hautverbrühungen (selten, bspw. durch heiße Wärmflaschen auf den Bauch) |
| - Hautmazerationen (meistens) |
| - Retikuläre Hautverfärbungen im Bereich der Haut „unter Wasser“ ( „water level lines“) (selten) |
| - Erhöhte Strom- oder Wasserrechnung (meistens) |
| *Komplikationen des langjährigen Cannabiskonsums* |
| - Körperlich: Refluxösophagitis, Gastroparese, Gastritis, Opstipation, Bronchitis. Psychisch: Entzugs- und Abhängigkeitssymptome inklusive Gedächtnis- und Exekutivleistungsstörungen (meistens); Panikattacken, Depression, Psychose oder Manie (häufig bis selten) |
| *Komplikationen von Kontaminationen* (z.B. Pflanzenschutzmittel, Schwermetalle, synthetische Cannabinoide) |
| - z.B. Somatitis, Hepatitis, Pankreatitis, Niereninsuffizienz, Myocarditis, Myocardinfarkt, Encephalitis, Vaskulitis, Myopathie, Neuropathie (*selten*) |
| *in schwerer Ausprägung (selten) mit dem Potential für maligne Herzrhythmustörungen, kardiogene Synkopen, Bewusstseinsstörungen, Delirien, provozierte epileptische Anfälle, Muskelschwäche und Ateminsuffizienz  ** in schwerer Ausprägung (selten) mit dem Potential für Orthostase-Reaktion, prä-renales Nierenversagen Bewusstseinsstörungen, Delirien, provozierte epileptische Anfälle und Thromboembolien |
