# Supplementary material for: The cannabinoid hyperemesis syndrome—A narrative review
Source: Nervenarzt. 2025 Jul 21;97(4):377–81. [Article in German] doi: 10.1007/s00115-025-01864-0 (PMC13314702; doi:10.1007/s00115-025-01864-0)
Supplement: Supplementary file 3 — eTabelle 3: Differenzialdiagnosen des CHS* [5, 15, 21, 24, 27] [file 115_2025_1864_MOESM3_ESM.docx]

eTabelle 3: Differentialdiagnosen des CHS* (5,15,21,24,27)

| - - - - Chronische Migräne (pz) |
| --- |
| - - - - Cyclic Vomiting Syndrom (CVS) (z) |
| - - - - Hyperemesisis gravidarum (pz während der Schwangerschaft) |
| - - - - Intoxikations- oder Entzugserscheinungen (pz) |
| - - - - Essstörungen (pz) |
| - - - - Psychogenes Erbrechen (pz) |
| - - - - Ruminations-Syndrom (pz) |
| - - - - Schädel-Hirn-Trauma |
| - - - - Herzerkrankungen (pz bei Angina pectoris) |
| - - - - Intra-abdominelle Erkrankungen (pz) |
| - - - - Gastroparese unabhängig vom Cannabis (z.B. bei Diabetes mellitus, anderen autonomen Neuropathien) |
| - - - - Erhöhter Hirndruck |
| - - - - Emetophilie |
| - - - - Stoffwechselerkrankungen (z.B. Porphyrie) |
| - - - - Endokrine Erkrankungen (z.B. M. Addison) |
| - - - - Mesenterialinfarkt, Arteria-mesenterica-superior-Syndrom |
| *geordnet nach abnehmender Wahrscheinlichkeit von oben nach unten bei unter 50-Jährigen mit einem positiven Cannabisnachweis im Urin und zyklischem (z) oder pseudozyklischem (pz, *scheinbar zyklischem*) Verlauf. Die anderen Differentialdiagnosen verlaufen eher nicht-zyklisch. |
