# Supplementary material for: The cannabinoid hyperemesis syndrome—A narrative review
Source: Nervenarzt. 2025 Jul 21;97(4):377–81. [Article in German] doi: 10.1007/s00115-025-01864-0 (PMC13314702; doi:10.1007/s00115-025-01864-0)
Supplement: Supplementary file 4 — eTabelle 4: Behandlungsempfehlung [5, 6, 13, 14, 21, 25] [file 115_2025_1864_MOESM4_ESM.docx]

eTabelle 4: Behandlungsempfehlung (5,6,13,14,21,25)

| A: **Akutmaßnahmen** |
| --- |
| - Flüssigkeits- und Elektrolyt-Substitution |
| - Bei refraktärer Übelkeit und Erbrechen 0.05 mg/kg Halperidol i.m. (maximal 5 mg*);* vorher Aufklärung die mögliche Entwicklung akuter Dystonien   *Cave: vorher EKG zum Ausschluss einer QTc-Verlängerung, bei QTc-Verlängerung keine Haloperidol-Gabe wegen besonderer Vorsicht bei noch vulnerablen Elektrolytverhältnissen bei schwerem Erbrechen; zunächst Ausgleich der Elektrolystörungen inklusive Kalium und Magnesium* |
| - Alternativ   zunächst heiße Dusche oder Bad in Betracht ziehen  *oder/und*  Capsaicin-Creme: bis zu 5 g 0.1% am Körperstamm (nach Korn et al (13) auch 1.Wahl) |
| - *zusätzlich*   Lorazepam 1mg oder 5-10 mg Diazepam i.v. (nach Korn et al (13) zur Beruhigung und dadurch ggf. zur Symptomlinderung) |
| - *zusätzlich*   *bei Refluxösophagitis/Gastritis: Protonenpumpenhemmer* |
| ***B. Psychoedukation*** |
| *- Empathische und vorurteilsfreie Gesprächsführung über den Cannabiskonsum (Berücksichtigung, dass die Betroffenen den Cannabiskonsum lieber verheimlichen wollen, Anbieten eines Drogentests)* |
| *- Aufklärung über die kausale Rolle von Cannabis und die anhaltende Cannabisabstinenz als einzige Möglichkeit zur Vollremission bzw. Heilung des CHS* |
| *- Motivationsgespräch zur Abstinenzeinleitung, Vermittlung an eine regionale Suchtberatungsstelle* |
